# Immunoproteomics based identification of thioredoxin reductase GliT and novel *Aspergillus fumigatus *antigens for serologic diagnosis of invasive aspergillosis

**DOI:** 10.1186/1471-2180-12-11

**Published:** 2012-01-18

**Authors:** Li-ning Shi, Fang-qiu Li, Mei Huang, Jing-fen Lu, Xiao-xiang Kong, Shi-qin Wang, Hai-feng Shao

**Affiliations:** 1 Laboratory of Molecular Biology, Institute of Medical Laboratory Sciences, Jinling Hospital, School of Medicine, Nanjing University, 305 East Zhongshan Road, Nanjing 210002, PR China

## Abstract

**Background:**

There has been a rising incidence of invasive aspergillosis (IA) in critically ill patients, even in the absence of an apparent predisposing immunodeficiency. The diagnosis of IA is difficult because clinical signs are not sensitive and specific, and serum galactomannan has relatively low sensitivity in this group of patients. Therefore, more prompt and accurate disease markers for early diagnosis are needed. To establish disease markers demands a thorough knowledge of fungal antigens which may be detected in the serum or other body fluids of patients. Herein we report novel immunodominant antigens identified from extracellular proteins of *Aspergillus fumigatus*.

**Results:**

Extracellular proteins of *A. fumigatus *were separated by two-dimensional electrophoresis (2-DE) and probed with the sera from critically ill patients with proven IA. The immunoreactive protein spots were identified by MALDI-TOF mass spectrometry (MALDI-TOF -MS). Forty spots from 2DE gels were detected and 17 different proteins were identified as immunogenic in humans. Function annotation revealed that most of these proteins were metabolic enzymes involved in carbohydrate, fatty acid, amino acid, and energy metabolism. One of the proteins, thioredoxin reductase GliT (TR), which showed the best immunoactivity, was analyzed further for secretory signals, protein localization, and homology. The results indicated that TR is a secretory protein with a signal sequence exhibiting a high probability for secretion. Furthermore, TR did not match any human proteins, and had low homology with most other fungi. The recombinant TR was recognized by the sera of all proven IA patients with different underlying diseases in this study.

**Conclusions:**

The immunoreactive proteins identified in this study may be helpful for the diagnosis of IA in critically ill patients. Our results indicate that TR and other immunodominant antigens have potential as biomarkers for the serologic diagnosis of invasive aspergillosis.

## Background

In recent decades, invasive aspergillosis (IA) has emerged as an important cause of morbidity and mortality in patients with prolonged neutropenia. However, several reports have recently described a rising incidence of IA in critically ill patients, even in the absence of an apparent predisposing immunodeficiency [1-6]. The incidence of IA in critically ill patients ranges from 0.3% to 5.8% [2,3,6], and carries an overall mortality rate > 80%, with an attributable mortality of approximately 20% [4,5]. Critically ill patients are prone to develop immunologic derangement, which renders them more vulnerable for *Aspergillus *infections. The risk factors for IA include chronic obstructive pulmonary disease (COPD) and other chronic lung diseases [1-4,7,8], prolonged use of steroids [2,9], advanced liver disease [2-4,10], chronic renal replacement therapy [11,12], near-drowning [4,13-15], and diabetes mellitus [2,3,9].

The diagnosis of such IA is difficult because signs and symptoms are non-specific. The conventional diagnostic methods, such as tissue examination and microbial cultivation, may lack sensitivity in the first stages of infection in critically ill patients. As a result, the diagnosis of IA is often established after a long delay or following autopsy. Currently, the best-characterized circulating marker used in the diagnosis of IA is galactomannan (GM), which is present in the cell walls of most *Aspergillus *species. The commercial Platelia *Aspergillus *assay (BioRad™, Marnes-La-Coquette, France) has been included in the EORTC/MSG criteria for probable IA. However, a recent meta-analysis indicated that GM testing is more useful in patients with prolonged neutropenia (sensitivity, 72%-82%) than in non-neutropenic, critically ill patients (sensitivity, 40%-55%) [16]. Further studies suggested that the host immune status may influence GM release. It appears that GM production is proportional to the fungal load in tissues [17]. Although neutropenic patients and non-neutropenic, critically ill patients are susceptible to IA, the pathology of the disease is quite different in these two groups of patients. In neutropenic patients and animal models, IA is characterized by thrombosis and hemorrhage from rapid and extensive hyphal growth [18]. However, in non-neutropenic, critically ill patients and animal models, IA is characterized by limited angioinvasion, tissue necrosis, and excessive inflammation [18,19]. The limited angioinvasion and low fungal load result in a low level of GM released by the fungus. The use of the GM assay for the diagnosis of IA in non-neutropenic patients is very limited. Therefore, more prompt and accurate disease markers for early diagnosis are needed, which requires a thorough knowledge of fungal antigens detected in the serum or other body fluids of infected patients.

*A. fumigatus *is the most common opportunistic pathogen that causes life-threatening IA in human beings. The ability of *A. fumigatus *to acquire and process growth substrates from its host is dependent on factors released from the fungi. The extracellular proteins of *A. fumigatus*, which are released during the germination of conidia and growth of hyphae, consist of secreted enzymes, toxins, and other secondary metabolites which are pathogenic and responsible for invasion of the structural barrier of the host [20]. Studies on the extracellular proteins of *A. fumigatus *and their immunogenic potential are therefore important for further understanding the pathogenesis of *A. fumigatus *and targets for the immunodiagnosis of the diseases. It is not surprising that some of the proteins may be major elicitors of specific immune responses, which could be brought into play to establish prognosis and develop new diagnostic procedures for IA.

We have recently observed that high levels of antibody against extracellular proteins of *A. fumigatus *are often present in the sera of critically ill patients with proven IA. This finding prompted us to discover the potential novel biomarkers for the diagnosis of IA in such patients. The investigation of specific antigens is strongly supported by the combination of immunoproteomics and bioinformatics. The completion of the genomes of *A. fumigatus *[21] and other *Aspergillus *species [22-25] makes it possible to identify the antigens of *Aspergillus *species on a global scale. In this study we searched for the immunodominant antigens from the crude culture filtrate using an immunoproteomic approach. As a result, a total of 17 immunodominant antigens were identified. One of the antigens, thioredoxin reductase GliT (TR), which showed the best immunoactivity, was cloned and expressed in *Escherichia coli*. Our results indicate that this protein could be useful for the early diagnosis of IA.

## Results

### Characterization of the patients

**Figure 1:**
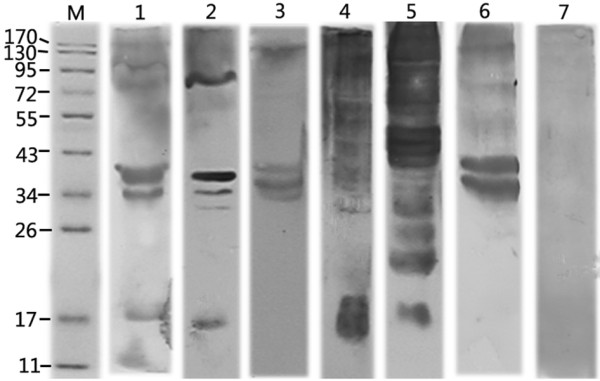
**Western blot analysis of *A. fumigatus *extracellular proteins and sera of proven IA patients**. Filtrate proteins (10 μg) of *A. fumigatus *during growth in YEPG medium at 37°C for 14 days were separated by SDS-PAGE and probed with sera from 6 patients with proven IA and control patients. Lane M, molecular weight marker; lanes 1-6, shows Western blot with sera from each of 6 proven IA patients; lane 7, shows Western blot with pooled sera of control patients.

Six patients with proven IA, and different underlying diseases and expressing high levels of anti-*Aspergillus *antibodies were selected for the immunoproteomic analysis. The details of the characteristics of the six patients with proven IA are listed in Table 1, histopathological results are given in Additional file 1 and the Western blots are shown in Figure 1. Multiple bands of immunogenic proteins were observed in each case, but not in the control sera. The enzyme-linked immunosorbent assay (ELISA) values of the patients with proven IA and the controls ranged from 1.105 to 2.561 and 0.114 to 0.362, respectively.

**Table 1:** Clinical characteristics of proven IA patients

Patient	Age (years)	sex	underlying disease	Immunosuppressant agents	Infected site	Patients outcome	Culture sample and result	Histo evidence
**1**	30	Female	systemic lupus eythematosus, lupus nephritis	Methylprednisolone	lung	Alive	Sputum, *A. fumigatus*	Percutaneous lung biopsy
**2**	39	Male	Shock, previously healthy	None	lung	Alive	BAL, *A. fumigatus*	Transbronchial biopsy
**3**	62	Male	DM, HP	None	lung	Dead	Sputum, *A. fumigatus*	Percutaneous lung biopsy + autopsy
**4**	44	Male	near-drowning	None	lung	Alive	BAL, *A. fumigatus*	Transbronchial biopsy
**5**	56	Female	Chronic obstructive pulmonary disease	Methylprednisolone	lung	Alive	BAL, *A. fumigatus*	Transbronchial biopsy
**6**	65	Male	renal transplantation	Prednisone, mycophenolate	lung	Alive	BAL, *A. fumigatus*	Transbronchial biopsy

Abbreviations: BAL = bronchoalveolar lavage

### Identified immunoreactive proteins

**Figure 2:**
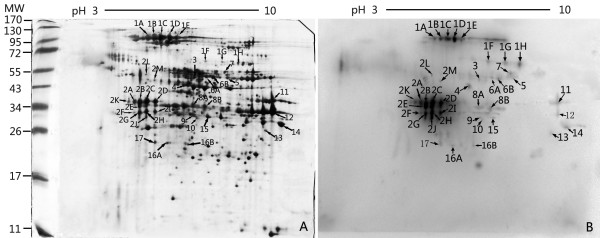
**2-DE analysis and Western blot for identification of immunogens from filtrate proteins of *A. fumigatus***. (**A**) 2-DE of filtrate proteins of *A. fumigatus *during growth in YEPG medum at 37°C for 14 days. (**B**) Immunoblot using pooled sera from proven IA patients. Filtrate proteins (150 μg) were separated by isoelectric focusing on Immobiline Dry strips (pH range, 3-10) followed by SDS-PAGE and silver staining. Standard molecular mass markers are indicated. Distinct protein spots (n = 39) with specific IgG immunoreactivity, as seen in corresponding immunoblots (**B**), were subjected to tryptic digestion followed by MALDI-TOF-MS analysis for identification (marked with arrow). The 17 proteins identified are numbered and listed in Table 2. Spot No. 2A-2 M was identified as thioredoxin reductase GliT.

The 2-DE and Western blot analyses of the filtrate proteins are shown in Figure 2. A total of 40 distinct immunoreactive spots were identified. The 39 successfully identified spots corresponded to 17 individual proteins. The sequence coverage ranged from 18%-70%, and the MASCOT scores were from 68 to 258. The identified proteins with molecular weights, isoelectric points, Mascot scores, and sequence coverage are listed in Table 2 (MS data of all immunoreactive spots identified are shown in Additional file 2). Several proteins occurred in multiple spots. Post-translational modifications are a likely explanation, resulting in altered molecular masses and/or isoelectric points. All 17 proteins are shown as a protein spot on the 2-DE gel and a corresponding immunogenic spot on the matching film. Of 17 identified proteins, 14 were matched with *A. fumigatus *(Af 293), and 3 showed homology to proteins from another *Aspergillus *species. Most of these proteins are metabolic enzymes that are involved in carbohydrate, fatty acid, amino acid, and energy metabolism. Seven of these proteins have been reported as antigens of *Aspergillus *and other fungi, and others have not been described as antigens before, such as fumarylacetoacetate hydrolase FahA, aldehyde dehydrogenase AldA, aromatic aminotransferase Aro8, G-protein comlpex beta subunit CpcB, actin cytoskeleton protein (VIP1), phytanoyl-CoA dioxygenase family, urate oxydase UaZ, 3-hydroxybutyryl-CoA dehydrogenase, proteasome component Pre8, putative and hypothetical protein. One protein of interest, which showed the best immunoreactivity, was identified as TR.

**Table 2:** Immunoreactive proteins of *A.fumigates *identified by MALDI-TOF-MS

**Spot no**.	Accession No. (GenBank)	Organism	Protein name	Peptides matched	Sequence coverage(%)	Mascot score	BLAST score (E-value)	Theoretical pI/Mr(kDa)	Probable functions
**1A-1H**	GI:71001112	*Aspergillus fumigatus *Af293	secreted dipeptidyl peptidase DppV	26	33	135	1.60E-08	5.59/79.7	Metabolism of dipeptides
**2A-2M**	GI:70992029	*Aspergillus fumigatus *Af293	thioredoxin reductase GliT	20	54	149	6.30E-10	5.44/36.2	Provide self-protection to *A. fumigatus*
**3**	GI:159123228	*Aspergillus fumigatus *A1163	FAD dependent oxidoreductase, putative	25	44	173	2.50E-12	5.94/51.5	Oxidoreductase
**4**	GI:70989411	*Aspergillus fumigatus *Af293	fumarylacetoacetate hydrolase FahA	13	37	85	0.0015	5.95/46.9	Phenylalanine catabolism, Tyrosine catabolism
**5**	GI: 119492487	*Neosartorya fischeri *NRRL 181	aspartyl aminopeptidase	20	40	98	8.90E-05	6.03/53.9	proteolysis, tissue invasion
**6A-6B**	GI: 70992355	*Aspergillus fumigatus *Af293	aldehyde dehydrogenase AldA	25	54	171	4.00E-12	6.30/61.4	Alcohol metabolism
**7**	GI: 71002030	*Aspergillus fumigatus *Af293	aromatic aminotransferase Aro8	19	52	145	3.10E-08	5.96/58.3	Aromatic aminoacid family metabolic process
**8A-8B**	GI: 70999466	*Aspergillus fumigatus *Af293	fructose-bisphosphate aldolase, class II	19	62	137	9.90E-09	5.55/39.9	Glycolysis, Carbohydrate degradation
**9**	GI: 119499942	*Neosartorya fischeri *NRRL 181	G-protein comlpex beta subunit CpcB	18	59	130	5.00E-08	6.06/35.3	Receptor signaling, intracellular signal transduction pathways, and protein synthesis
**10**	GI: 71001310	*Aspergillus fumigatus *Af293	actin cytoskeleton protein (VIP1)	13	40	86	0.0013	5.93/28.3	Component of cytoskeleton
**11**	GI: 159129975	*Aspergillus fumigatus *A1163	phytanoyl-CoA dioxygenase family	15	64	109	6.30E-06	6.08/33.7	Oxidization
**12**	GI: 70988713	*Aspergillus fumigatus *Af293	pectate lyase A	13	44	96	0.00014	6.23/33.8	Carbohydrate metabolism, cell wall biogenesis/degradation
**13**	GI: 71001408	*Aspergillus fumigatus *Af293	urate oxydase UaZ	12	32	80	0.0052	7.24/34.1	Metabolism of urate
**14**	GI: 70986899	*Aspergillus fumigatus *Af293	malate dehydrogenase, NAD-dependent	23	70	258	7.90E-21	9.08/35.8	Cellular carbohydrate metabolic process
**15**	GI: 169764553	*Aspergillus oryzae *RIB40	hypothetical protein	13	41	92	2.90E-04	6.21/35.3	unknown
**16A-16B**	GI: 70982195	*Aspergillus fumigatus *Af293	3-hydroxybutyryl-CoA dehydrogenase	15	44	90	0.00052	6.33/36.0	Fatty acid metabolic process
**17**	GI: 121711615	*Aspergillus clavatus *NRRL 1	proteasome component Pre8, putative	12	44	80	0.0052	5.55/30.1	Proteolysis involved in cellular protein catabolic process

### Bioinformatics analysis of TR

TR was predicted as a secretory protein with the presence of signal sequences with good predictive value (signalP probability, 0.808). The protein localization of TR was predicted using WoLF PSORT, and the result also indicated that this protein might be an extracellular protein (Query Protein WoLFPSORT prediction: extr, 12.0; cyto, 6.5; cyto_nucl, 4.0; mito, 3.0; pero, 2.0). This protein was BLAST-searched for sequence homology with human proteins and other fungi using the BLAST program (http://www.ncbi.nlm.nih.gov/BLASTp). The results indicated that TR of *A. fumigatus *had no matches with human proteins. Furthermore, TR of *A. fumigatus *had low homology with other fungi, such as *Candida albicans *(25%), *C. tropicalis *(25%), *C. glabrata *(24%), *C. guilliermondii *(27%), *C. dubliniensis *(23%), *Saccharomyces cerevisiae *(24%), *Cryptococcus neoformans *(28%), and *Penicillium marneffei *(27%). This protein was also BLAST-searched for sequence homology with all protein databases using the Uniprot program (http://www.uniprot.org). The results indicated that TR of *A. fumigatus *has < 55% homology with all proteins in the databases, excluding pyridine nucleotide-disulphide oxidoreductase of *A. fischeri *(identitiy, 94%) and the putative uncharacterized protein of *A. terreus *(identitity, 80%). TR of *A. fumigatus *also had low homology with most other *Aspergillus *species, such as *A. oryzae *(55%), *A. flavus *(54%), *A. nidulans *(50%), *A. clavatus *(47%), and *A. niger *(41%), as shown in Additional file 3.

### Expression and antigenicity of TR recombinant protein

**Figure 3:**
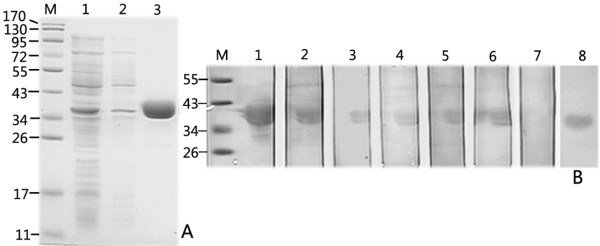
**SDS-PAGE and Western blot analysis of the recombinant thioredoxin reductase GliT (TR) of *A. fumigatus***. (**A**): SDS-PAGE analysis of the recombinant TR expressed in *Escherichia coli *BL21. Lane M, molecular weight marker; lane 1, pET28a -TR in *E. coli *BL21, 1 mM isopropyl-β- D - thiogalactoside induced for 5 h; lane 2, pET28a-TR in *E. coli *BL21, not induced; lane 3, purified recombinant TR; (**B**): Western blot analysis of the purified recombinant TR with sera of 6 patients with proven IA, pooled control patients, and monoclonal mouse anti-His antibody. lanes 1-6, Western blot of purified recombinant TR with sera from 6 patients with proven IA; lane 7, Western blot of purified recombinant TR with sera of pooled control patients; lane 8, Western blot of purified recombinant TR with monoclonal mouse anti-His antibody.

After induction by isopropyl-β-D-thiogalactoside (IPTG), the recombinant 6-His-tagged TR was expressed, and a novel protein band corresponding to 36 kDa was detected by SDS-PAGE (Figure 3A). Most of the recombinant proteins were soluble. After purification using a TALON metal affinity resin, the protein purity was approximately 91%. Protein identity was unambiguously confirmed by MALDI-TOF MS, whereas following tryptic digestion proteins were identified yielding 37% sequence coverage (the MS spectra are shown in Additional file 4). Western blot showed that the recombinant proteins could be recognized by the sera from all six patients with proven IA (Figure 3B).

## Discussion

*Aspergillus fumigatus *is known as the most common cause of IA in humans. The extracellular proteins of *A. fumigatus*, which functions in enabling the fungi to adhere to host tissues and take up nutrients from the hosts, play an important role in the interaction between fungi and hosts. The extracellular location of these proteins enables the proteins to interact easily with the host immune system. Accordingly, studies on the immunogenic nature of these extracellular proteins are of particular importance to understand the pathogenesis of *A. fumigatus*. The immunogenic proteins may represent candidate markers for the diagnosis of IA. In fact, preparations of *A. fumigatus *extracellular proteins have been used to detect antibodies in the sera of human patients or experimentally infected animals, and culture filtrates have also been used to raise polyclonal antibodies to detect *A. fumigatus *antigens in the sera or urine of patients or experimentally infected rats [26,27]. Our group has previously observed that high titers of antibodies against extracellular proteins of *A. fumigatus *are often present in the sera of critically ill IA patients (unpublished data). However, knowledge of the extracellular proteins of *A. fumigatus *and the corresponding antibodies is limited. To investigate the immunodominant antigens, the extracellular proteins at different intervals were extracted from 4 media (*Aspergillus *minimal medium, YEPG, Czapek-Dox medium, and RPMI-1640), then probed with sera of IA patients. The results indicated that the protein yield reached a maximum at 14 days, and the YEPG culture supernatant contained the maximum number of proteins reacting with the sera in comparison to other media (unpublished data). Thus, the 14-day YEPG filtrate proteins were used in a subsequent study.

In the present study, the immunodominant proteins from the culture filtrate of *A. fumigatus *were detected using an immunoproteomic approach. The immunoreactive protein spots showing a significantly different pattern of recognition in sera from IA patients when compared with specimens from controls were characterized by MS. Of 17 identified proteins, 7 have been reported as antigens of *Aspergillus *and/or other fungi. For example, DppV, TR, FAD-dependant oxygenase, pectate lyase A, aspartyl aminopeptidase, and NAD-dependent malate dehydrogenase are already known as antigens or allergens of *Aspergillus *[28-31]. Fructose-bisphosphate aldolase was identified as an immunogen in patients with systemic candidiasis [32]. Furthermore, diverse groups have reported that some metabolic enzymes interact specifically with human extracellar matrix proteins, such as fibronectin, laminin, and integrin-like vitronectin [33,34], and are involved in adhesion and pathogenesis.

The immunodominant nature of these proteins is valuable for the diagnosis of invasive fungal infections. DppV, a member of the dipeptidyl-peptidase family in *A. fumigatus*, is identical to one of the principal antigens used in the diagnosis of IA. Moreover, DppV can generate protection responses, and improve the survival rate of *Aspergillus*-infected mice [28]. DppV can also bind with collagen or other human proteins and degrade them, which can damage the host. Recombinant DppV has shown a great potential in the serodiagnosis of IA in immunocompromised and immunocompetent patients [35]. NAD-dependent malate dehydrogenase, a key enzyme in glycometabolism that catalyze the reversible conversion between malate and oxaloacetate, was reported recently as an allergen of *A. fumigatus *and *A. versicolor *[29]. Malate dehydrogenase was also shown to be a *Paracoccidioides brasileinsis *immunogenic protein [36] as well as a *Candida albicans *immunogen [32]. Aspartyl aminopeptidase, an enzyme that specifically degrades only amino-terminal acidic amino acids from peptides, was recently reported as an antigen of *A. fumigatus *[30] .

TR of *A. fumigatus *has been described as an extracellular antigenic protein by two recent studies [30,31]. In one former study, the secreted fraction of two geographically different strains (190/96 and DAYA) of *A. fumigatus *were used to identify new immunogenic molecules reacting with pooled ABPA patient sera (IgG and IgE). TR was only detected on 2DE immunoblots of the secreted proteome of the DAYA strain probed with the IgE antibody fraction from pooled ABPA patients sera [31]. This result suggested that TR might not be a good biomarker for ABPA. In another study, the immunosecretome of *A. fumigatus *was detected using pooled patient sera (total n = 22 patients [ABPA, n = 11; aspergilloma, n = 5; IA, n = 6]). The immunoreactive intensity of TR was lower than most other proteins [30]. A possible explanation is that the anti-TR antibody titers were not high in pooled sera because most cases included in the study were not IA.

Although investigators in other laboratories recently noted the antigenic nature of TR [30,31], no study has found shown diagnostic value for TR in non-neutropenic patients with IA. We showed that TR (spot no. 2A-2 M) had the strongest immunoreactivity with patient sera. TR, a component of the gliotoxin biosynthetic cluster, provides self protection to *A. fumigatus *against gliotoxin [37,38]. This protein has been described as an extracellular protein of *A. fumigatus *by Singh and Kumar [30,31]. However, Schrettl et al. showed that GliT is preferentially localized in the cytoplasm and nuclei by a GFP-GliT construct [38]. To predict whether or not GliT is actively secreted into the culture supernatant, we used two bioinformatic tools (SignalP and WoLF PSORT) to analyze its localization. Our results support the findings of Singh and Kumar [30,31]. Homology analysis indicated that TR had no match with human proteins, as Kumar et al. [35] reported. Muro et al. [39] also reported that the fungal TR has only 19% sequence similarity to human TR. Furthermore, sequence homology analysis showed that TR of *A. fumigatus *has low homology with most other *Aspergillus *species as well as most other fungi. Therefore, TR could be considered as a specific antigen of *A. fumigatus *and as a potential biomarker for the serological diagnosis of IA. In order to study its diagnostic potential, we cloned the TR gene and purified the recombinant protein. Immunoblots showed that recombinant protein could be recognized by the sera from all six IA patients. These results suggested that the TR of *A. fumigatus *could be developed as a biomarker for the diagnosis of IA, especially in critically ill patients.

One of the strengths of our study was that all patients included had histopathologic evidence and positive cultures. This enabled us to discriminate between invasive disease and colonization. However, we do realize that the study design has limitations. We did not further investigate the reactivity of individual patient serum with the extracellular fraction of *A. fumigatus*, thus we cannot provide data whether or not these proteins consistently react with individual IA patient serum. Moreover, the cases used in this study were limited in number, therefore the diagnostic value of the antigen identified should be validated in further prospective studies using large-scale serum specimens.

## Conclusions

*Aspergillus fumigatus *is known to be the most common opportunistic pathogen that causes life-threatening IA in humans. The ability of *A. fumigatus *to acquire and process growth substrates from its host is dependent on the factors the fungus releases. Studies on the extracellular proteins of *A. fumigatus *and their immunogenic potential are therefore important for further understanding the pathogenesis of *A. fumigatus *and targets for the immunodiagnosis of the diseases. Our study has highlighted the immunodominant antigens of extracellular proteins. A total of 17 proteins of *A. fumigatus *were identified as antigens in humans. Some of the proteins have been reported as antigens of *Aspergillus *and/or other fungi. Interestingly, our study revealed the best immunoactive protein, TR, which showed great potential for the diagnosis of IA.

## Materials and methods

### Patients and control subjects

Serum samples expressing high titers of antibodies against the extracellular proteins of *A. fumigatus *were obtained from six non-neutropenic-proven IA patients with different underlying diseases. All serum samples were obtained at the time of diagnosis. Two-to-four samples were obtained sequentially per patient. Sera from 20 ICU patients without clinical or microbiological evidence of IA, including 8 patients with chronic obstructive pulmonary disease, 6 patients with chronic renal disease, 3 patients with renal transplantation, and 3 patients with acute pancreatitis (age range, 33-75 years), were used as negative controls. Two samples were obtained per control patient (one during early admission and one before leaving the hospital). *Aspergillus*-specific IgG antibodies in the sera of all patients were determined by an indirect ELISA using filtrate proteins of *A. fumigatus *(1 μg/ml) as the coating antigen (sera diluted 1:1000). All sera were stored at -70°C. Sera of IA patients and controls were pooled separately for immunoproteomics analysis. According to EORTC-MSG criteria, proven IA refers to histopathologic evidence of tissue invasion by septated, acutely-branching filamentous fungi, together with a positive culture (sputum and/or bronchoalveolar lavage) [39]. The study protocol was approved by the Ethics Committee of the hospital and informed consent was obtained from all patients included in the study.

### Preparation of extracellular proteins

*A. fumigatus *(strain CMCC (f) A1a) was obtained from the Microbial Culture Collection Management Committee of China, Medical Mycology Center. The fungus was first grown on Sabouraud agar plates at 37°C for 3 days. The conidia were collected and incubated in yeast-extract-peptone-glucose (YEPG) broth (1% yeast extract, 2% peptone, and 2% glucose) in a 500-ml flask on a shaker at 37°C for 14 days. Then, the culture supernatant was collected by filtration. The proteins were recovered by trichloroacetic acid (TCA) precipitation, as described previously [40]. Finally, the precipitates were resuspended in two-dimensional electrophoresis (2-DE; 7 M urea, 2 M thiourea, 4% [w/v] CHAPS, 1% [w/v] DTT, 1% protease inhibitor cocktail [v/v], and 2% [v/v] IPG buffer [pH 3-10]) lysis buffer, and stored at -70°C. The protein concentration was determined by the Bradford method using BSA as the standard.

### Two-dimensional electrophoresis and Western blot analysis

Samples containing 150 μg of filtrate protein were separated by 2-DE, as described elsewhere [41], using immobilized, non-linear pH 3-10 gradient strips (24 cm; Amersham Biosciences, Uppsala, Sweden) for isoelectric focusing, and 12.5% sodium dodecylsulfate polyacrylamide gels for the second dimension separation. All gels were silver-stained according to published procedures [42] or electrotransferred to polyvinylidene fluoride (PVDF) membranes [43]. Three replicates were run for each sample.

Western blot was performed as described previously [44]. Briefly, the membranes were probed with primary antibody (pooled sera of patients with proven IA and pooled control sera [1:1000 dilution in each case]) at 4°C overnight. Subsequently, the membranes were thrice washed with Tris-buffered saline (pH 7.5) containing 0.05% (v/v) Tween-20 (TBST) for 10 min and incubated with horseradish peroxidase (HRP)-conjugated goat anti-human IgG (1:2000 dilution) for 2 h at room temperature. The membranes were then washed with TBST and the signal was detected with an enhanced chemiluminescence detection kit (Amersham Biosciences, Uppsala, Sweden). The autoradiographs were developed using Kodak imaging films according to the instrument of the manufactorer. Three replicates were performed for each sample.

### Protein identification and database searches

The specific immunoreactive protein spots were matched through overlapping images of the blot and gel. The Western blots were matched first with their own Ponceau stain images, then were compared with the silver-stained gel. Subsequently, the spots of interest were excised from the 2DE gels for tryptic in-gel digestion and matrix-assisted laser desorption ionization time-of-flight mass spectrometry (MALDI-TOF-MS) on a time-of-flight Ultraflex II mass spectrometer (Bruker Daltonics, Bremen, Germany). The peak lists of each protein spot were searched against the NCBI database using Mascot (v2.1.03; Matrix Sciences, London, UK). The following search parameter criteria were used: significant protein MOWSE score at a *p *< 0.05; minimum mass accuracy, 100 ppm; 1 missed cleavage site allowed (cysteine carbamidomethylation, acrylamide-modified cysteine, and methionine oxidation); similarity of pI and relative molecular mass specified; and minimum sequence coverage of 15%.

### Bioinformatics analysis of TR

The signal peptide and the probability of TR were predicted using SignalP software (http://www.cbs.dtu.dk/services/SignalP/). Another subcellular localization prediction tool, WoLF PSORT (http://www.wolfpsort.org), was used to analyze the amino acid sequences of proteins for prediction of cellular localization. Homology analysis was performed using the BLAST program (http://www.ncbi.nlm.nih.gov/BLASTp and http://www.uniprot.org).

### Expression, purification, and Western blot analysis of recombinant thioredoxin reductase GliT

For RNA preparation, 100 mg of frozen mycelium was ground under nitrogen and the whole RNA was extracted using Trizol (Invitrogen, USA). cDNA was generated using AMV reverse transcriptase (Promega, Madison, WI, USA). The TR gene was amplified using the following primers: 5'-CACACATATGTCGATCGGCAAACTAC-3' and 5'-ACTGAATTCCTATAGCTCCTGATCGAGACG-3'. The resulting 1005-bp fragments were cloned into the pET-28a (+) expression vector (Novagen, Germany). The TR sequence was 100% identical to the gene of *A. fumigatus *strain Af293. Then, the recombinant His_6_-TR was expressed in *E. coli *BL21 competent cells, and purified using a TALON metal affinity resin (Clontech, Japan). Fractions containing the purified TR were pooled, dialyzed against 0.1 M phosphate buffered saline (PBS; pH 7.2), and stored at -70°C. Protein identity of the recombinant TR was confirmed by MALDI-TOF MS.

Western blot of the purified recombinant proteins was carried out as described earlier. Monoclonal mouse anti-HIS antibody (diluted 1:4000), the serum samples from six patients with proven IA, and pooled sera from healthy individuals (diluted 1:1000) were used as primary antibodies. HRP-rabbit anti-mouse IgG (1:5000) and HRP-goat anti-human IgG (diluted 1:2000) were used as secondary antibodies. The antibody-bound proteins were then visualized using the DAB kit (Maixin_Bio, China).

## Abbreviations

MALDI-TOF: matrix-assisted laser desorption/ionization-time of flight.

## Competing interests

The authors declare that they have no competing interests.

## Authors' contributions

FQL conceived, coordinated and designed the study. LNS contributed to the acquisition, analysis and interpretation of data and drafted the manuscript. XXK, SQW and JFL performed the experiment and were involved in drafting the article. MH and HFS participated in sample collection and data acquisition. All the authors have read and approved the final manuscript.

## Supplementary Material

Additional file 1 **Histopathological results of 6 proven IA patients**. This figure shows the histopathological section of lung tissues obtained from 6 proven IA patients exhibiting *Aspergillus *with septated and acutely-branching hyphae. Click here for file

Additional file 2 **MS-based identification of all immunoreactive protein of *A. fumigatus *during growth in YEPG medium at 37°C for 14 days**. This table lists all MS-identified proteins that were marked in Figure 2. Click here for file

Additional file 3 **BLAST search of *A. fumigatus *thioredoxin reductase Glit in UniProtKB**. This table lists 1000 BLAST results. Click here for file

Additional file 4 **MS spectra of the recombinant thioredoxin reductase Glit**. Protein identity of the recombinant thioredoxin reductase Glit was confirmed by MALDI-ToF MS whereby peptides (following tryptic digestion) were identified yielding 13 peptides matched and 37% sequence coverage. Click here for file
