# Supplementary figures and images for: Immunoproteomics based identification of thioredoxin reductase GliT and novel Aspergillus fumigatus antigens for serologic diagnosis of invasive aspergillosis
Source: BMC Microbiol. 2012 Jan 18;12:11. doi: 10.1186/1471-2180-12-11 (PMC3398318; doi:10.1186/1471-2180-12-11)

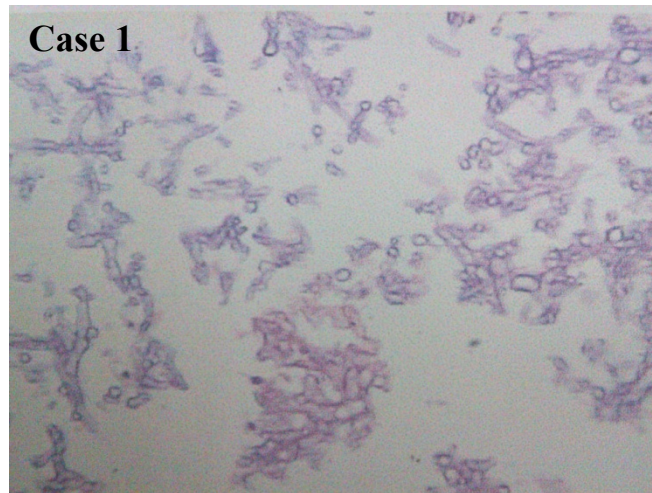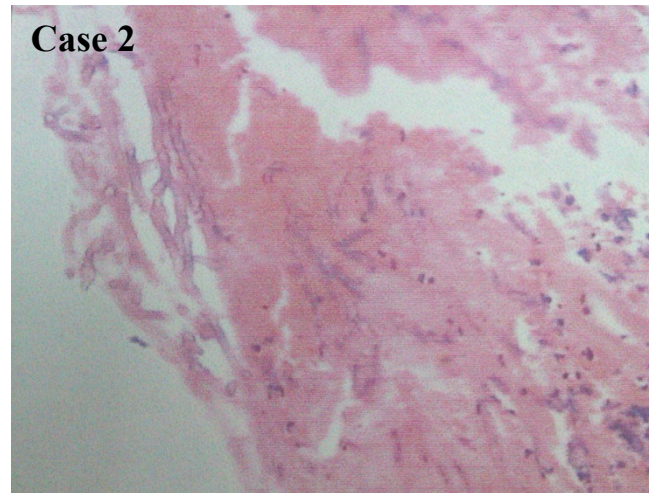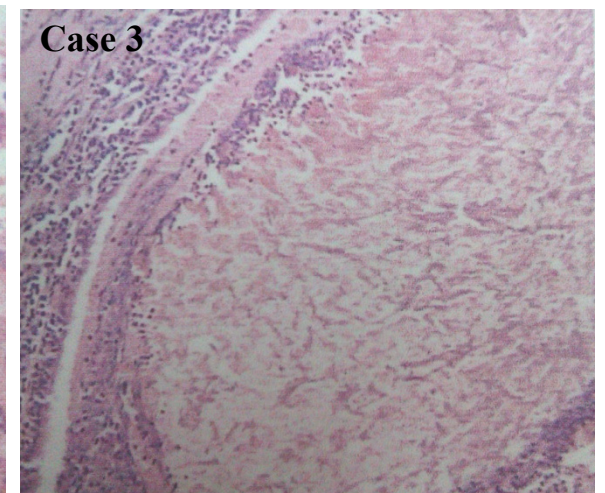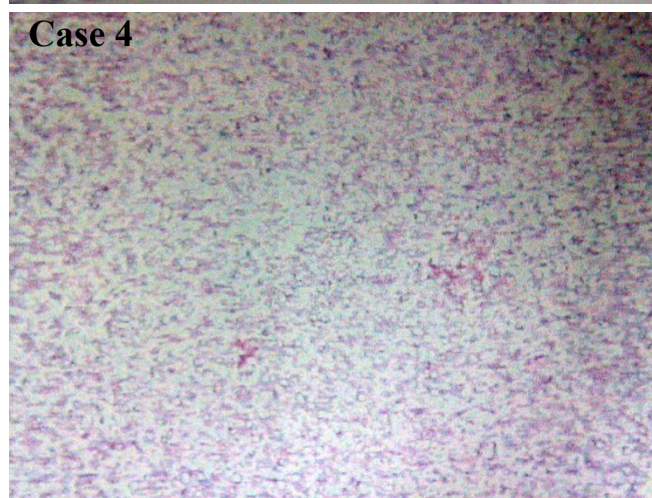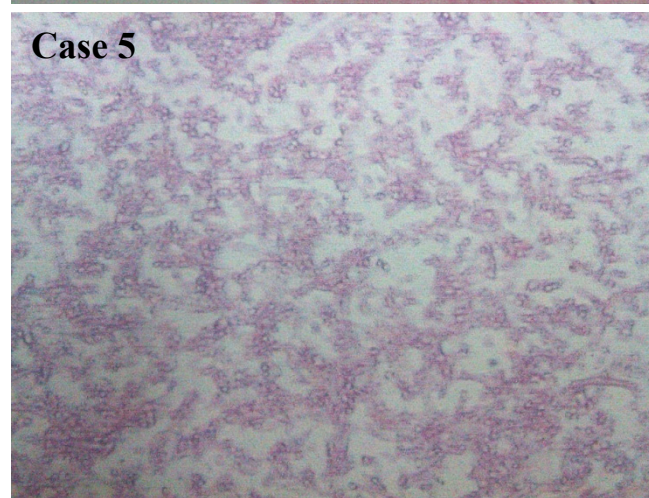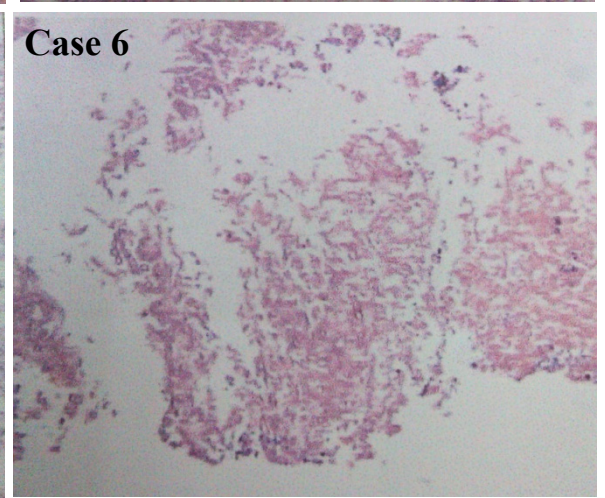

**Additional file 1: Histopathological results of 6 proven IA patients**

Supplement: Additional file 1 — Histopathological results of 6 proven IA patients. This figure shows the histopathological section of lung tissues obtained from 6 proven IA patients exhibiting Aspergillus with septated and acutely-branching hyphae. [file 1471-2180-12-11-S1.PDF]

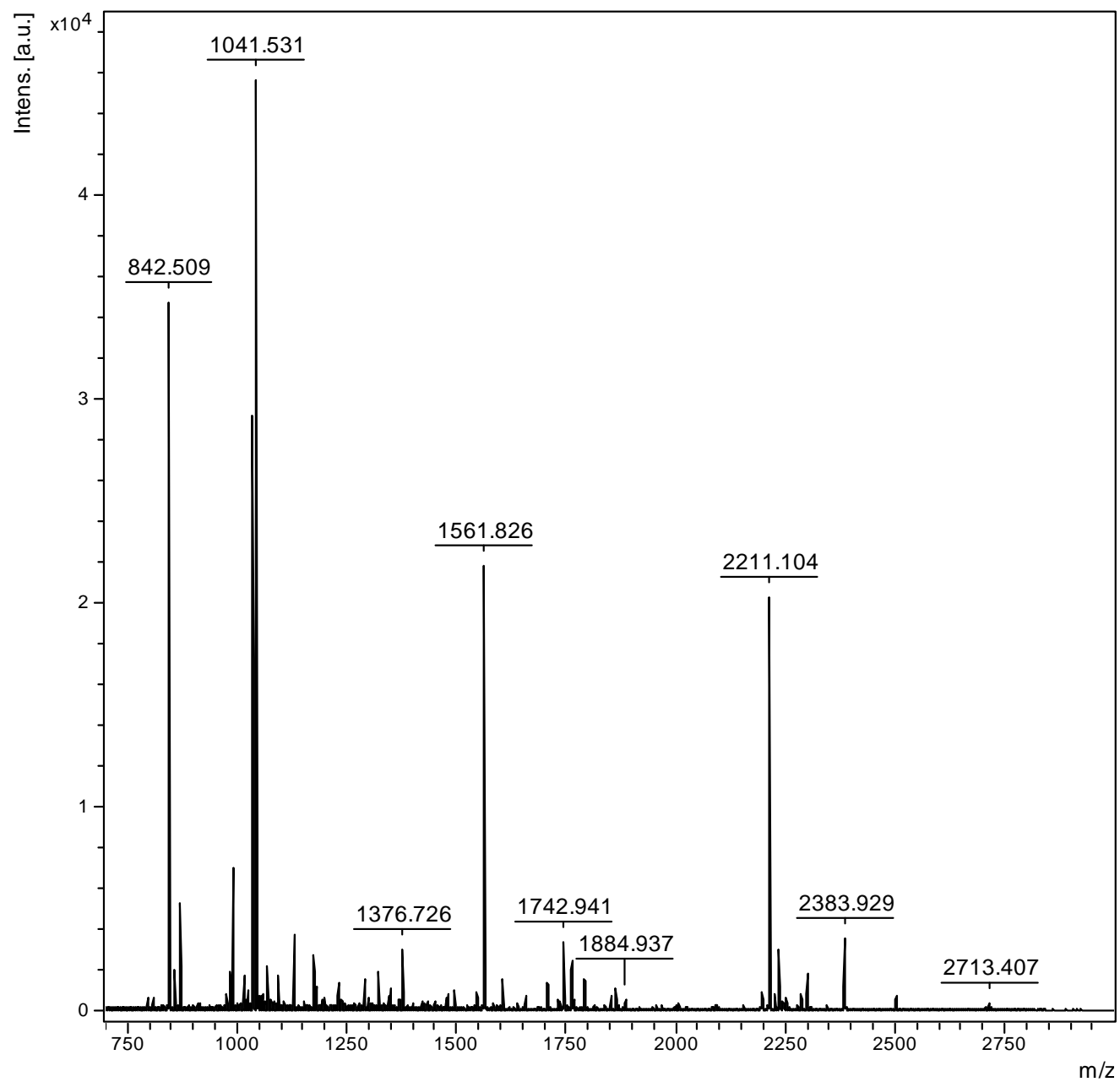

**Additional file 4: MS spectra of the recombinant thioredoxin reductase Glit.**

Supplement: Additional file 4 — MS spectra of the recombinant thioredoxin reductase Glit. Protein identity of the recombinant thioredoxin reductase Glit was confirmed by MALDI-ToF MS whereby peptides (following tryptic digestion) were identified yielding 13 peptides matched and 37% sequence coverage. [file 1471-2180-12-11-S4.PDF]
